# Glycolipid Antibodies in Patients With Chronic Idiopathic Axonal Polyneuropathy and Gluten Neuropathy

**DOI:** 10.1111/ene.70422

**Published:** 2025-11-03

**Authors:** Panagiotis Zis, Artemios Artemiadis, Susan Halstead, Georgios M. Hadjigeorgiou, Hugh Willison, Marios Hadjivassiliou

**Affiliations:** ^1^ Medical School University of Cyprus Nicosia Cyprus; ^2^ Institute of Infection, Immunity and Inflammation, College of Medical, Veterinary and Life Sciences University of Glasgow Glasgow UK; ^3^ Sheffield Institute of Gluten Related Disorders (SIGReD) Sheffield Teaching Hospitals NHS Foundation Trust Sheffield UK

**Keywords:** antibodies, autoimmunity, gluten, glycolipids, polyneuropathy

## Abstract

**Objective:**

To evaluate the presence of IgM and IgG antibodies in the serum of patients with chronic idiopathic axonal polyneuropathy (CIAP) and gluten neuropathy (GN) using a newly developed microarray technique.

**Methods:**

Sera from 42 CIAP patients, 32 GN patients, and 79 healthy controls were tested for IgM and IgG antibodies against 16 single glycolipids and their 120 heteromeric complexes. Positivity rates were analyzed using the 95th percentile from controls.

**Results:**

IgM positivity against 8 heteromeric complexes was more frequent in CIAP patients (16.7% to 28.6%) compared to controls, while GN patients showed higher rates (18.8% to 46.9%) across 15 complexes. GN patients had a higher rate of IgM positivity against the GM2:GT1b complex than CIAP patients (46.9% vs. 21.4%). IgG antibodies against seven antigens were also more prevalent in CIAP (16.7% to 26.2%) and GN patients (18.8% to 28.1%) than controls. GN patients showed a higher rate of IgG positivity against the GM1:Sulfatide complex than CIAP patients (15.6% vs. 0%).

**Conclusions:**

This is the first study to report increased antibody detection rates against peripheral nerve glycolipids, both individually and in complexes. The findings suggest that a significant proportion of CIAP and GN patients may have an autoimmune pathogenesis, potentially targetable by immunotherapy.

## 1 Introduction

Chronic idiopathic axonal polyneuropathy (CIAP) and gluten neuropathy (GN) are two distinct types of peripheral neuropathy (PN) that share similar clinical and electrophysiological characteristics [1, 2]. CIAP by definition describes axonal neuropathies of insidious onset, with slow or no progression and no etiology being identified despite appropriate investigations [3]. GN occurs in patients with celiac disease (CD) or serologically confirmed gluten sensitivity (GS), with no other etiologies. It responds excellently to a strict gluten‐free diet (GFD), slowing or stopping progression [2].

Autoimmunity plays a significant role in the pathogenesis of GN, evidenced not only by the presence of antibodies against gliadin and/or transglutaminase, but also by inflammatory changes seen in sural nerve biopsies [4]. Previous research suggests an autoimmune component in the pathogenesis of CIAP [5, 6], however the exact pathophysiology remains poorly understood. Shedding light on the role of autoimmunity and identifying specific peripheral nerve immune markers in routine clinical practice remains challenging. Obtaining deeper insights into the pathogenesis might in the future enable more targeted immune therapies for certain subgroups of patients.

Recently, Halstead et al. introduced a novel microanalytical method for detecting antibodies against multiple antigenic targets in patients with autoimmune neuropathies [7]. The rationale behind this approach is that autoantibodies targeting glycolipids in vivo may behave differently than those tested in laboratory assays. To address this, they created a multi‐array platform featuring single glycolipids and all their combinational heteromeric glycolipid complexes.

In this study, we applied the same microanalytical method, using an extended array of 136 antigens (16 single and 120 heteromeric glycolipid complexes) to evaluate IgM and IgG antibodies in 42 CIAP patients, 32 GN patients, and 79 healthy controls in an attempt to shed light on the role of autoimmunity in these conditions.

## 2 Methods

Patients with CIAP and GN and healthy controls were recruited from the Sheffield Institute of Gluten‐Related Diseases, Sheffield Teaching Hospitals NHS Trust, UK and the University of Cyprus. All participants were over 18 years old and provided written informed consent. GN patients were required to have a diagnosis of PN based on nerve conduction studies (NCS), serological evidence of GS (positive IgA or IgG antigliadin, anti‐endomysial and/or anti‐transglutaminase antibodies) prior to starting a GFD, and no other potential causes. CIAP patients were required to present with symptoms and signs of PN, with confirmation of axonal damage in NCS, after ruling out other potential causes. The exact battery of tests needed before making a diagnosis of GN and CIAP has been published elsewhere [3].

All GN patients were tested for serological evidence of GS at the time of recruitment. Patients without detectable serological markers were considered to be adhering successfully to a strict GFD.

All patients were classified as having either a length‐dependent axonal peripheral neuropathy (LD) or a sensory ganglionopathy (SG) based on their NCS.

The fabrication of the antigen array, sera screening, and scanning methodology has been previously described elsewhere [7]. The analyses were performed at the University of Glasgow. Statistical analysis included descriptive statistics for the study sample. Patients were categorized based on their IgG and IgM autoantibody status into two groups: positive or negative, determined by the 95th percentile of antibody titers from the healthy control group. Group comparisons were made by using the chi‐square test with continuity correction or Fisher exact test. Partial correlations between age and antibody titres, controlling for disease status, were conducted where appropriate. The level of significance was set at 0.05. Statistical analysis was performed by using IBM SPSS Statistics for Windows, Version 21.0. Armonk, NY: IBM Corp.

## 3 Results

The study sample included 42 CIAP patients (26 females, mean age 72.8 ± 9.0 years), 32 GN patients (25 females, mean age 68.8 ± 9.9 years), and 79 healthy controls (32 females, mean age 60.8 ± 9.5 years). The majority of patients had LD—81% in the CIAP group and 75% in the GN group. At the time of recruitment, 14 GN patients (44%) were adhering to GFD.

Table 1 presents the results for significant group differences in IgM antibodies. A higher proportion of IgM‐positive patients (combined CIAP and GN) were found for 11 heteromeric complexes compared to controls. Specifically, IgM positivity against 8 heteromeric complexes was significantly more frequent in CIAP patients than in controls, with positivity rates ranging from 16.7% to 28.6%. For GN patients, significant IgM positivity was found against 15 heteromeric complexes, with positivity rates ranging from 18.8% to 46.9%. A significant difference between CIAP and GN was seen in only 1 of 136 antigens; GN patients were significantly more likely to show IgM positivity against the GM2:GT1b complex(46.9% vs. 21.4%).

**TABLE 1:** Significant serum positivity rates and group comparisons of 74 patients (42 CIAP and 32 GN patients) and 79 healthy controls for IgM antibodies against single and heteromeric antigenic complexes.

	Patients	Healthy controls	Sig.^a^		CIAP	Healthy controls	Sig.^a^
GM2:GD1a	15 (20.3%)	4 (5.1%)	0.009	GM2:GT1a	8 (19%)	4 (5.1%)	0.023
GM2:GT1a	16 (21.6%)	4 (5.1%)	0.005	GM2:GT1b	9 (21.4%)	4 (5.1%)	0.011
GM2:GT1b	24 (32.4%)	4 (5.1%)	< 0.0001	GM2:GD3	7 (16.7%)	4 (5.1%)	0.047
GM2:GQ1b	16 (21.6%)	4 (5.1%)	0.005	PS:GT1b	12 (28.6%)	4 (5.1%)	0.001
GM2:GD3	14 (18.9%)	4 (5.1%)	0.016	GM4:GT1b	8 (19%)	4 (5.1%)	0.023
PS:GT1b	20 (27%)	4 (5.1%)	< 0.0001	GA1:GT1a	11 (26.2%)	4 (5.1%)	0.002
GM4:GT1b	14 (18.9%)	4 (5.1%)	0.016	GT1a:GT1b	8 (19%)	4 (5.1%)	0.023
GA1:GT1a	24 (32.4%)	4 (5.1%)	< 0.0001	GT1b:GalC	7 (16.7%)	4 (5.1%)	0.047
GT1a:GT1b	15 (20.3%)	4 (5.1%)	0.009				
GT1a:Sulphatide	12 (16.2%)	4 (5.1%)	0.047				
GT1b:GalC	15 (20.3%)	4 (5.1%)	0.009				

	GN	Healthy controls	Sig.^a^		CIAP	GN	Sig.^a^
GM2:GD1a	10 (31.3%)	4 (5.1%)	0.001	GM2:GT1b	9 (21.4%)	15 (46.9%)	0.039
GM2:GT1a	8 (25%)	4 (5.1%)	0.005				
GM2:GT1b	15 (46.9%)	4 (5.1%)	< 0.0001				
GM2:GQ1b	10 (31.3%)	4 (5.1%)	0.001				
GM2:GD3	7 (21.9%)	4 (5.1%)	0.013				
GM2:SGPG	6 (18.8%)	4 (5.1%)	0.032				
PS:GT1b	8 (25%)	4 (5.1%)	0.005				
PS:GQ1b	7 (21.9%)	4 (5.1%)	0.013				
PS:GD3	7 (21.9%)	4 (5.1%)	0.013				
GM4:GT1b	6 (18.8%)	4 (5.1%)	0.032				
GA1:GT1a	13 (40.6%)	4 (5.1%)	< 0.0001				
GT1a:GT1b	7 (21.9%)	4 (5.1%)	0.013				
GT1a:Sulphatide	6 (18.8%)	4 (5.1%)	0.032				
GT1b:GQ1b	6 (18.8%)	4 (5.1%)	0.032				
GT1b:GalC	8 (25%)	4 (5.1%)	0.005				

^a^ Chi‐square test with continuity correction for 2 × 2 tables, or Fisher exact test. Level of significance *p* < 0.05.

Regarding IgG autoantibodies, seven antigens showed increased positivity in patients (combined CIAP and GN) compared to controls (Table 2). Specifically, IgG antibodies against eight antigens were significantly more prevalent in CIAP patients compared to controls, with positivity rates ranging from 16.7% to 26.2%. Similarly, IgG antibodies against seven antigens were significantly more frequent in GN patients than in controls, with positivity rates ranging from 18.8% to 28.1%. A significant difference between CIAP and GN was seen in only 1 of 136 antigens; GN patients were significantly more likely to show IgG positivity against the GM1:Sulfatide complex than CIAP patients(15.6% vs. 0%).

**TABLE 2:** Significant serum positivity rates and group comparisons of 74 patients (42 CIAP and 32 GN patients) and 79 healthy controls for IgG antibodies against single and heteromeric antigenic complexes.

	Patients	Healthy controls	Sig.^a^		CIAP	Healthy controls	Sig.^a^
GD1b	12 (16.2%)	4 (5.1%)	0.047	GD1b	7 (16.7%)	4 (5.1%)	0.047
SGPG	17 (23%)	4 (5.1%)	0.003	SGPG	8 (19%)	4 (5.1%)	0.023
Sulphatide	17 (23%)	4 (5.1%)	0.003	Sulph	9 (21.4%)	4 (5.1%)	0.011
GM1:PS	14 (18.9)	4 (5.1%)	0.016	GM1:PS	9 (21.4%)	4 (5.1%)	0.011
GM2:PS	17 (23%)	4 (5.1%)	0.003	GM2:PS	11 (26.2%)	4 (5.1%)	0.002
PS:Sulphatide	12 (16.2%)	4 (5.1%)	0.047	PS:GD1b	7 (16.7%)	4 (5.1%)	0.047
GalC:Sulphatide	14 (18.9%)	4 (5.1%)	0.016	PS:Sulphatide	7 (16.7%)	4 (5.1%)	0.047
				GalC:Sulphatide	7 (16.7%)	4 (5.1%)	0.047

	GN	Healthy controls	Sig.^a^		CIAP	GN	Sig.^a^
PS	6 (18.8%)	4 (5.1%)	0.032	GM1:Sulphatide	0 (0%)	5 (15.6%)	0.013
GT1b	6 (18.8%)	4 (5.1%)	0.032				
GQ1b	7 (21.9%)	4 (5.1%)	0.013				
SGPG	9 (28.1%)	4 (5.1%)	0.002				
Sulph	8 (25%)	4 (5.1%)	0.005				
GM2:PS	6 (18.8%)	4 (5.1%)	0.032				
GalC:Sulphatide	7 (21.9%)	4 (5.1%)	0.013				

^a^ Chi‐square test with continuity correction for 2 × 2 tables, or Fischer exact test. Level of significance *p* < 0.05.

Regarding all antigens that showed significantly higher positivity, only a very weak correlation between the titer of IgM GT1a:Sulph and IgM PS:GT1b and age (correlation coefficient 0.184 and 0.178 respectively) was found on partial correlation analysis. No correlation between the titer of all other IgM and IgG antigens and age was found.

Regarding IgM and IgG antigens that showed significantly increased positivity no differences were seen between LD and SG patients in both the CIAP and GN groups.

Comparing the antibody profiles that showed increased positivity between GN patients who were adherent to a GFD and those who were not, we found that none of the IgM antigens showed significant differences between the two subgroups. Among the IgG antigens, a significant difference was observed in the GM2:PS complex, with 0% of patients on a GFD testing positive, compared to 33.3% of patients not adhering to the diet (*p* = 0.024).

## 4 Discussion

This study is the first to demonstrate that a significant proportion of patients with CIAP and GN exhibit elevated IgM or IgG serum antibodies against various single and heteromeric glycolipid complexes in comparison to controls. The fact that the patient groups differed significantly in antibody positivity for only 1 out of 136 IgM and IgG antigens further supports the hypothesis that CIAP, like GN, may be driven by underlying autoimmune mechanisms.

Previous research on CIAP found abnormal antibody panels in up to 10% of patients [8]. However, in the present study, positivity rates were higher, reaching 28.6% for IgM antibodies against the PS:GT1b complex and 26.2% for IgG antibodies against the GM2:PS complex. This difference may, in part, be attributed to our use of a broader and more comprehensive antiglycolipid panel, which included both single and complex antigens—potentially increasing the sensitivity for detecting relevant antibody reactivity. However, we must acknowledge that our sample size is relatively small, and a sampling effect cannot be ruled out.

Our findings should be interpreted with caution given some further limitations. First, the method we used is new and requires validation through future research. Positivity in our study was assessed using the 95th percentile of titers from a group of healthy controls. Expanding the sample size of healthy controls in future studies would help refine these cut‐off values. Second, while glycolipid complexes can mimic in vivo environments and the conformations of glycolipid epitopes, the method is not cell‐based. Nonetheless, this technique enables the detection of antibodies against a wide array of antigens—136 in this study—which would be impractical using cell‐based methods. Assessing antibodies against numerous antigens increases the risk of false‐positive results due to statistical variability and cross‐reactivity. This issue could be mitigated by analyzing patterns of autoantibody positivity using statistical methods like dimension reduction with principal component analysis. However, our study's small sample size limited the feasibility of such approaches and could lead to misinterpretation. Finally, in this study we did not include IgA antibody detection. IgA‐mediated autoimmunity is particularly relevant in CD but in extra‐intestinal manifestations IgG and IgM are more relevant particularly as the majority of GN don't have CD. Including IgA antibody detection in a future study could provide additional insights, particularly in subgroups of patients.

Despite these limitations, this study provides novel evidence that the pathogenesis of CIAP and GN involves a significant immune component. Future research should validate these findings by using ELISA analysis, larger cohorts and refine the microanalytical method further. Including an additional comparison group of patients with neuropathy due to causes not typically associated with autoimmunity—such as diabetic or chemotherapy‐induced neuropathy—could further strengthen the findings by helping to distinguish disease‐specific autoimmune responses from nonspecific background reactivity. Laboratory studies could also elucidate the precise role of anti‐glycolipid antibodies in the pathogenesis of these diseases. Importantly, identifying patients with autoimmunity‐related PN may open avenues for new, targeted immunological treatments.

## Conflicts of Interest

The authors declare no conflicts of interest.

## Data Availability

The data that support the findings of this study are available from the corresponding author upon reasonable request.

## References

[1] P. Zis , P. G. Sarrigiannis , A. Artemiadis , V. Skarlatou , and M. Hadjivassiliou , “Chronic Idiopathic Axonal Polyneuropathy: Electrophysiological Progression and Human Leukocyte Antigen Associations,” Muscle & Nerve 63, no. 4 (2021): 567–571, 10.1002/mus.27164. 33440030

[2] P. Zis , P. Sarrigiannis , A. Artemiadis , D. S. Sanders , and M. Hadjivassiliou , “Gluten Neuropathy: Electrophysiological Progression and HLA Associations,” Journal of Neurology 268, no. 1 (2021): 199–205, 10.1007/s00415-020-10137-6. 32754830

[3] P. Zis , P. G. Sarrigiannis , D. G. Rao , C. Hewamadduma , and M. Hadjivassiliou , “Chronic Idiopathic Axonal Polyneuropathy: A Systematic Review,” Journal of Neurology 263, no. 10 (2016): 1903–1910, 10.1007/s00415-016-8082-7. 26961897

[4] M. Hadjivassiliou , R. A. Grünewald , R. H. Kandler , et al., “Neuropathy Associated With Gluten Sensitivity,” Journal of Neurology, Neurosurgery, and Psychiatry 77, no. 11 (2006): 1262–1266, 10.1136/jnnp.2006.093534. 16835287 PMC2077388

[5] R. K. Gherardi , J. P. Farcet , A. Créange , et al., “Dominant T‐Cell Clones of Unknown Significance in Patients With Idiopathic Sensory Neuropathies,” Neurology 51, no. 2 (1998): 384–389, 10.1212/wnl.51.2.384. 9710007

[6] P. Kelkar , W. R. McDermott , and G. J. Parry , “Sensory‐Predominant, Painful, Idiopathic Neuropathy: Inflammatory Changes in Sural Nerves,” Muscle & Nerve 26, no. 3 (2002): 413–416, 10.1002/mus.10202. 12210372

[7] S. K. Halstead , G. Kalna , M. B. Islam , et al., “Microarray Screening of Guillain‐Barré Syndrome Sera for Antibodies to Glycolipid Complexes,” Neurol Neuroimmunol Neuroinflamm 3, no. 6 (2016): e284, 10.1212/NXI.0000000000000284. 27790627 PMC5055300

[8] G. I. Wolfe , W. H. El‐Feky , J. S. Katz , W. W. Bryan , F. H. Wians , and R. J. Barohn , “Antibody Panels in Idiopathic Polyneuropathy and Motor Neuron Disease,” Muscle & Nerve 20, no. 10 (1997): 1275–1283, 10.1002/(sici)1097-4598(199710)20:10<1275::aid-mus10>3.0.co;2-2. 9324084

